# Screening for Y Chromosome Microdeletion in a Nonobstructive Azoospermic Male Patient with Allogeneic Bone Marrow Transplantation from His Sister

**DOI:** 10.1155/2010/541061

**Published:** 2010-12-16

**Authors:** Hakan Gurkan, Faruk Kucukdurmaz, Tolga Akman, Filiz Aydın, Ates Kadioglu

**Affiliations:** ^1^Department of Medical Biology, Istanbul Faculty of Medicine, Istanbul University, 34390 Istanbul, Turkey; ^2^Section of Andrology, Department of Urology, Istanbul Faculty of Medicine, Istanbul University, 34390 Istanbul, Turkey

## Abstract

Genomic DNA of a patient diagnosed with nonobstructive azoospermia and with the history of allogenic bone marrow transplantation from his sister due to chronic myeloid leukemia was isolated from peripheral blood in order to screen Y chromosome microdeletions. 13 short tagged sites belonging to AZF a, b, and c loci were detected with multiplex polymerase chain reaction technique. Bands were determined in ZFX/ZFY wells, whereas no bands were determined in wells of other STS regions. DNA isolation was done from buccal mucosa smear to obtain genomic DNA from patient's own cells and multiplex polymerase chain reaction technique was performed again. Bands were seen in all wells of 13 STS regions. Y chromosome microdeletion was not detected in the patient. In conclusion, genomic DNA isolation in patients undergoing BMT should be done from patients' own cells.

## 1. Introduction

Infertility affects approximately 10–15% of couples, and male factor infertility represents almost 50% of cases [1]. Among infertile men about 40–50% have azoospermia and severe oligozoospermia. Genetic abnormalities may cause infertility by affecting sperm production or sperm transport. These abnormalities are numerical (e.g., trisomy) and structural (e.g., inversions or translocations) chromosomal abnormalities, Y chromosome microdeletions, and gene mutations (e.g., cystic fibrosis). The frequencies of genetic abnormalities vary between 10–15% in oligozoospermic and azoospermic patients [2]. Because of this high prevalence, it is important to investigate the chromosomal abnormalities in these patients.

## 2. Case Report

A healthy 27-year-old man with normal phenotype was referred for the evaluation of infertility. In his physical examination, right testis was 10 cc, left testis was 8 cc with grade 1 varicocele. Vasa deferentia were palpable on both sides. Consecutive two semen analyses of the patient revealed azoospermia with normal ejaculate volume. His serum FSH was 24.29 mIU/mL (1.6–12.4 mIU/mL) and total testosterone was 495 ng/dL (260–1600 ng/dL). The patient with the diagnosis of nonobstructive azoospermia (NOA) had a history of allogeneic bone marrow transplantation (BMT) from his sister due to chronic myeloid leukemia (CML). Karyotyping and Y chromosome microdeletion analysis, which are necessary for the evaluation of NOA, were done with peripheral blood leucocytes and buccal mucosa samples because of the probable change in peripheral blood leucocytes after allogeneic bone marrow transplantation. There was no family history of infertility.

The patient, who had the diagnosis of CML with positive Philadelphia (Ph) translocation in February 1999, was treated with Hydroxyurea 500 mg/day for two years. The patient was conditioned with cyclophosphamide 60 mg/kg for two days and TBI with a total dose of 12 Gy in 3 fractionated doses before BMT. In 2001, allogeneic BMT was performed. After allogeneic BMT, the patient was treated with 26 mg methotrexate and 30 gr IVIG. In cytogenetic analysis performed on cultured cells from bone marrow material before allogeneic BMT, no metaphases were examined. After BMT, cytogenetic analysis revealed 6 metaphases examined by trypsin GTG banding, and 4 of them had 46,XX chromosomal constitution.

## 3. Materials and Methods

Molecular cytogenetic and Y chromosome microdeletion analysis was performed according to standard methods on cultured cells from the patient's peripheral blood. Genomic DNA was isolated in accordance with kit protocol (High pure PCR template preparation kit, Roche). In the laboratory, kit which is used for determination of Y chromosome microdeletion is certified with IVD (in vitro diagnostic). 13 STS (sequence tagged site) regions on Y chromosome (ZFX/ZFY, SRY, AZFa: sY84, sY86, DFFRY, DBY, AZFb: sY117, sY125, sY127, sY134, AZFc: DAZ gene sY254, sY255) were analysed by using 3 different polymerase chain reaction (PCR) mixes (Genequality AZF MX, AB-Analitica). Multiplex PCR reaction was performed with isolated genomic DNA samples in conformity with kit protocols. PCR products were subjected to 85 V for electrophoresis during 2 hours and 30 minutes in 3% mucinous agarose gel.

## 4. Results

**Figure 1:**
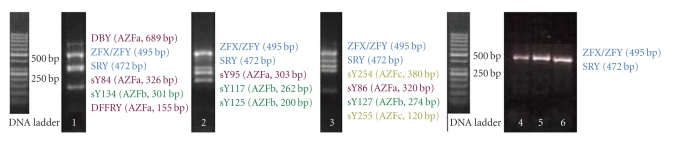
Photos of 3% high-resolution agarose (mucinous agarose) gel electrophoresis of the three multiplex PCR amplicons from the patient's DNA (genomic DNA was isolated from venous blood cells) and control DNA. Band patterns of the control DNA: 1, 2, and 3, band patterns of the patient's DNA: 4, 5, and 6.

**Figure 2:**
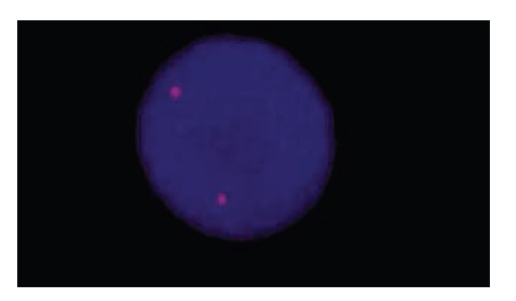
FISH analysis of interphase lymphocyte from the patient with DNA probes of CepX/Y.

**Figure 3:**
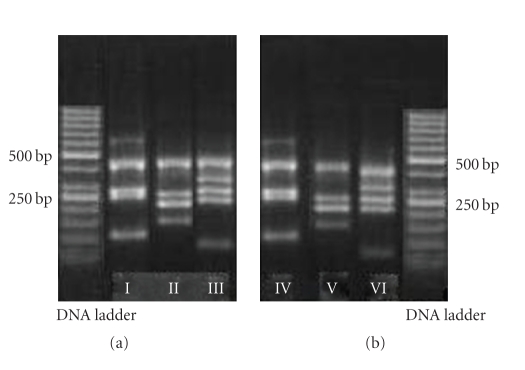
Photos of 3% high-resolution agarose (mucinous agarose) gel electrophoresis of the three multiplex PCR amplicons from the patient's DNA (genomic DNA was isolated from *buccal mucosa*) and control DNA. Band patterns of the control DNA: I, II, and III, band patterns of the patient's DNA: IV, V, and VI.

**Figure 4:**
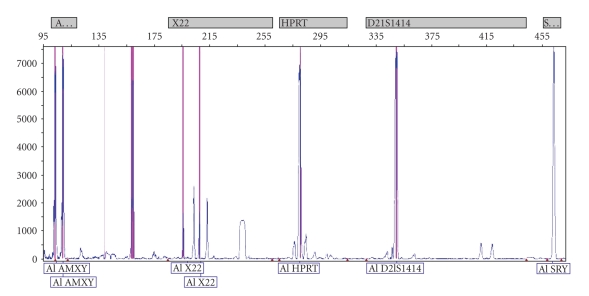
Electrophoretogram showing normal XY male sex chromosome constitution. Both the X- and Y- specific products of the AMXY (X-Y amelogenin gene) are present with a normal ratio of 1 : 1. The XY male sex chromosome constitution is confirmed by the occurrence of the SRY product. In this example, the presence of two sex chromosomes is also further confirmed by the normal heterozygous pattern of both pseudoautosomal markers X22.

When the results of electrophoresis were investigated with UV transilluminator, band patterns were detected only in ZFX/ZFY wells of all 3 PCR mixes. There was no band pattern in wells of other 12 STS regions (Figure 1). This situation was not compatible with phenotype of patient and could occur due to allogenic BMT from his sister. Therefore, chimerism analysis was done with FISH using CepX/Y DNA probe (Vysis) in order to indicate chimerism rate of sex chromosomes in cells of the peripheric circulatory system of the patient. The CEP X/Y DNA probe is a mixture of a SpectrumOrange labeled CEP X DNA probe (Xp11.1-q11.1 Alpha Satellite DNA) and a SpectrumGreen labeled CEP Y DNA probe specific for the alpha satellite centromeric region of chromosome X and the satellite III (Yq12) region of chromosome Y. Two orange signals of Xp11.1-q11.1 Alpha Satellite DNA were found on the interphase chromosome in 200 cells of peripheric circulatory system of patient. It was determined that chimerism rate was 100% XX (Figure 2). Towards this result, genomic DNA was isolated from buccal mucosa smear in order to obtain the patient's own cells. Isolated genomic DNA was evaluated with NanoDrop spectrophotometer (NanoDrop, Thermo Scientific, USA) to detect the density of this sample. 1 *μ*L DNA of patient was defined as 11.06 ng and rate of A260/A280 was defined as 1.42 which means that this amount was enough for PCR. Then, PCR was performed in accordance with kit protocol that was used previously. PCR products were subjected to 85 V for electrophoresis during 2 hours and 30 minutes in 3% mucinous agarose gel. Bands in wells of 3 different mixes including 13 STS regions were detected with UV transilluminator. There were band patterns in all wells, and Y chromosome microdeletion was not detected in patient (Figure 3). Sex chromosomes of patient were evaluated in terms of aneuploidy with multiplex PCR in conformity with kit protocol (Aneufast-QF PCR) used with Quantitative Fluorescent PCR (QF-PCR) technique. The QF-PCR Kit contains six multiplex marker sets of short tandem repeats (STRs) that can be used for amplification of selected microsatellites and the Amelogenin-SRY. PCR products were subjected to capillary electrophoresis in automatized device of DNA strand analysis (ABI-3100 Avant). Fragment analysis showed that the patient had XY chromosomal constitution (Figure 4). After PCR, AZF a, b, and c loci that were localized on Y chromosome long arm and did not generate recombination with X chromosome in meiosis were amplified (in the agarose gel, band pattern was positive in wells of 10 STS regions special to AZF loci). This also excludes the situation that patient could have XX sex chromosome in the beginning.

## 5. Discussion

The incidence of Y chromosome microdeletions was reported 10–15% and 5–10% in azoospermic and oligozoospermic patients, respectively [3].

In the literature, there is only one report defining a male with the diagnosis of CML and 46,XX chromosomal constitution [4]. That patient, like the present case, had bilateral small testes, azoospermia, and hypergonadotropic hypogonadism. Cytogenetic and molecular analysis has demonstrated that male XX is caused by at least three mechanisms, abnormal Y-X interchange (90%), genes other than testes-determining factor (TDF), and mosaicism [5]. It is anticipated that malignant disease itself and adjuvant or neoadjuvant regimens used for the treatment may result in oligozoospermia and azoospermia [6]. Thachil et al. reported that cancer itself has negative effects on fertility before application of any treatment modality [7]. It is also reported that chemotherapeutic agents used in the treatment of CML, like cyclosphosphamide, hydroxyurea, busulphan, chlorambucil, and radiotherapy, have deleterious effects on germ cells which may result in azoospermia [8, 9]. 

The reason of azoospermia in the present case in unclear whether it may be due to CML itself or the treatment regimens used to cure the disease.

In another study, it is reported that the rate of recovery of spermatogenesis was 17% in patients conditioned with cyclophosphamide combined with total body irradiation (TBI) before BMT. Recovery of spermatogenesis never occurred before the fourth year after transplantation and was observed up to 9 years. The incidence of azoospermia in those patients was 70.3% [10]. The rate of gonadal dysfunction in patients treated with cyclophosphamide with a cumulative dose of less than 400 mg/kg was less than 10% whereas this rate was increased up to 30% in prepubertal and 68–95% in adult patients treated with a cumulative dose of more than 400 mg/kg cyclophosphamide [9, 10].

During TBI, the diffused testicular dose is estimated to be 1-2% of the total dose applied. Direct irradiation (0.15–0.35 Gy) causes oligozoospermia; doses between 0.35 Gy and 0.5 Gy cause reversible azoospermia. Doses of 1.2 Gy are associated with a reduced risk of recovery of spermatogenesis. Time to recovery is also likely to depend on the dose [8]. Cumulative doses of fractionated radiotherapy of >2.5 Gy generally result in prolonged and likely permanent azoospermia. Radiation doses to the germinal epithelium of the testis given in 3- to 7-week fractionated courses cause more gonadal damage than single doses. An irreversible damage of the spermatogenesis will begin at a cumulative dose of >2.5 Gy when the radiation is fractionated [11]. The present case was conditioned with cyclophosphamide 60 mg/kg for two days and TBI with a total dose of 12 Gy in 3 fractionated doses before BMT. Recovery of spermatogenesis never occurred during the 8-year followup after BMT. 

Pretreatment sperm cryopreservation, although not applied in this case, should be offered to young, male cancer patients to prevent infertility due to the therapeutic regimens which causes oligozoospermia or azoospermia.

To the best of our knowledge, the study does not provide a new information methodologically but from the point of clinical view this is the first NOA case with the diagnosis of CML and 46,XX chromosomal constitution due to allogeneic BMT from his sister. In conclusion, genomic DNA investigation in patients undergoing BMT should be done with patients' own cells (buccal mucosa, tissue biopsy, etc.) to obtain accurate results about the chromosomal constitutions of these patients.
